# Diabetes among tuberculosis patients and its impact on tuberculosis treatment in South Asia: a systematic review and meta-analysis

**DOI:** 10.1038/s41598-021-81057-2

**Published:** 2021-01-22

**Authors:** Sanju Gautam, Nipun Shrestha, Sweta Mahato, Tuan P. A. Nguyen, Shiva Raj Mishra, Gabriele Berg-Beckhoff

**Affiliations:** 1 Faculty of Health Science, University of Southern Denmark, Odense, Denmark; 2 Community Health Development Nepal, Kathmandu, Nepal; 3 Nepal Development Society, Bharatpur, Nepal; 4 Unit for Health Promotion Research, University of Southern Denmark, Odense, Denmark

**Keywords:** Type 2 diabetes, Tuberculosis

## Abstract

The escalating burden of diabetes is increasing the risk of contracting tuberculosis (TB) and has a pervasive impact on TB treatment outcomes. Therefore, we conducted this systematic review and meta-analysis to examine the burden of diabetes among TB patients and assess its impact on TB treatment in South Asia (Afghanistan, Bangladesh, Bhutan, Maldives, Nepal, India, Pakistan, and Sri Lanka). PubMed, Excerpta Medica Database (EMBASE), and CINAHL databases were systematically searched for observational (cross-sectional, case–control and cohort) studies that reported prevalence of diabetes in TB patients and published between 1 January 1980 and 30 July 2020. A random-effect model for computing the pooled prevalence of diabetes and a fixed-effect model for assessing its impact on TB treatment were used. The review was registered with PROSPERO number CRD42020167896. Of the 3463 identified studies, a total of 74 studies (47 studies from India, 10 from Pakistan, four from Nepal and two from both Bangladesh and Sri-Lanka) were included in this systematic review: 65 studies for the prevalence of diabetes among TB patients and nine studies for the impact of diabetes on TB treatment outcomes. The pooled prevalence of diabetes in TB patients was 21% (95% CI 18.0, 23.0; *I2* 98.3%), varying from 11% in Bangladesh to 24% in Sri-Lanka. The prevalence was higher in studies having a sample size less than 300 (23%, 95% CI 18.0, 27.0), studies conducted in adults (21%, 95% CI 18.0, 23.0) and countries with high TB burden (21%, 95% CI 19.0, 24.0). Publication bias was detected based on the graphic asymmetry of the funnel plot and Egger’s test (p < 0.001). Compared with non-diabetic TB patients, patients with TB and diabetes were associated with higher odds of mortality (Odds Ratio (OR) 1.7; 95% CI 1.2, 2.51; *I2* 19.4%) and treatment failure (OR 1.7; 95% CI 1.1, 2.4; *I2* 49.6%), but not associated with Multi-drug resistant TB (OR 1.0; 95% CI 0.6, 1.7; *I2 *40.7%). This study found a high burden of diabetes among TB patients in South Asia. Patients with TB-diabetes were at higher risk of treatment failure and mortality compared to TB alone. Screening for diabetes among TB patients along with planning and implementation of preventive and curative strategies for both TB and diabetes are urgently needed.

## Introduction

Tuberculosis (TB) is the largest infectious disease killer in the world^1^. The South Asian region which consists of eight low and middle-income nations, namely Afghanistan, Bangladesh, Bhutan, India, Maldives, Nepal, Pakistan, and Sri Lanka alone shares for nearly 44% of the world TB cases^1^ and a high burden of TB mortality (681,975 deaths), 38% of the worldwide burden^2^. The high burden of TB is further intricated by the growing prevalence of various risk factors such as acquired immunodeficiency syndrome, kidney disease, malnutrition, and diabetes in South Asia; and are further compounded by the health system-related and patient related factors such as access, diagnosis and treatment completion^3,4^.

The burden of cardiometabolic diseases, particularly diabetes has become a major health problem in South Asian countries, with an expected rise in diabetes prevalence of more than 151% between 2000 and 2020^5,6^. Moreover, some studies have indicated that South Asians are at higher risk of developing cardiometabolic diseases including diabetes compared to other ethnic groups^5,7^. Diabetes has also been found to escalate the risk of contracting TB by three folds^8,9^. Likewise, diabetes is associated with a higher risk of failure in TB treatment or relapse, failure in culture conversion at 6-months and 2-months^10^ and deaths in TB patients, more precisely pulmonary TB patients^11^. Besides, a systematic review showed that there is an increased risk of mortality (RR = 1.89 (95% CI 1.52–2.36), combined outcome failure and death (RR = 1.69 (95% CI 1.36–2.12), and relapse (RR = 3.89 (95% CI 2.43–6.23) among patients with TB-diabetes comorbidity than TB only patients^12^. It is therefore foreseeable that this surge in the diabetes prevalence will increase vulnerability to TB infection and negative treatment outcomes among those with active TB disease^13–15^. The exact mechanism of how diabetes comorbidity impact health outcomes in TB patients has not been elucidated yet. However, there is some evidence for the negative impact of diabetes comorbidity on the TB treatment outcome^10,11,16^. Several mechanisms have been postulated for the diabetes impact on the TB treatment outcome that includes altered immunological response^17,18^, increased insulin resistance due to anti-tuberculosis drugs particularly, Rifampicin and impaired immunity due to diabetes itself^19^. However, evidence summarizing the impact of diabetes on TB treatment outcomes from the South Asian population is limited.

World Health Organization member countries have agreed upon an ambitious target to achieve 25% reduction in TB incidence and 75% reduction in TB mortality between 2015 and 2025, and 90% reduction in TB incidence and 95% reduction in TB mortality by 2035^20^. This ambitious target cannot be achieved unless the escalating burden of risk factors such as diabetes is properly addressed. Some systematic reviews have summarized the burden of diabetes among TB patients at the global and regional levels^3,9^. Previous systematic review by Noubiap et al. which reported global prevalence of diabetes among TB patients, did not provide comprehensive evidence from South Asian countries; failing to include many studies from India and reporting no studies from Afghanistan, Bhutan, Maldives and Nepal^3^. Therefore, a much larger study with updated information on both epidemiology and impact of TB-diabetes is urgently needed in the South Asia region.

This study will, therefore, examine the existing literature on the prevalence of diabetes and its impact on treatment outcomes among TB patients which can inform policymakers in devising strategies for integrated care of TB and diabetes at the national and regional levels.

## Results

### Study selection

Of the 3463 articles retrieved (3295 articles for estimating the prevalence of diabetes among TB patients and 168 articles for assessing the impact of diabetes among TB treatment outcome), 65 articles were found eligible for assessing the prevalence of diabetes among TB patients and nine for assessing the impact of diabetes on TB treatment outcomes as shown in the figures. (Supplementary Fig. 1). Included studies were observational studies conducted in at least one of the South Asian Association for Regional Cooperation (SAARC) countries in any age group or any gender and have reported prevalence of diabetes among tuberculosis patients or provide data that allowed computation of the impact of DM on tuberculosis treatment outcome.

### Study characteristics

The main characteristics of the studies included in the review are summarized in Supplementary Table 2. In total, 65 studies with 49,792 TB patients were included in the pooled prevalence for diabetes in tuberculosis patient, out of which 47 studies were from India^13,21–67^, 10 from Pakistan^68–77^, four from Nepal^78–81^ and two studies each from Bangladesh^82,83^ and Sri-Lanka^84,85^. However, there were no studies from Bhutan, Maldives, and Afghanistan. Almost all studies used consecutive sampling (73.8%), were hospital-based (93.8%), and collected data prospectively (76.9%). Regarding study design, two were case–control studies, 20 were of cohort design and the rest were cross-sectional studies. The sample size of studies varied from 101 to 8109 and the mean age of the participants (TB patients) ranged from 29–53.3 years. All studies included both the male and female participants and most of the studies included only adult TB patients (84.6%), while the remaining 10 studies (15.4%) included both adult and children TB patients as their participants. In a larger proportion of studies, diabetes was diagnosed by measuring blood glucose levels (random, fasting, and/or 2-h OGTT) (86.2%) while in the remaining studies (13.8%) the diagnosis was based on self-reporting, records, treatment cards and use of antidiabetic medications. Similarly, most of the studies used medical records, chest radiography, and sputum smear microscopy for identifying TB patients.

### Assessment of risk of bias

The risk of bias of the included studies was evaluated using the Newcastle Ottawa scales (NOS) adapted from Alebel et al.^9^ with slight modification. Only one-third of the studies (34) were judged to have a low risk of bias. Most studies were judged at a high risk of bias for item number three of the selection category description of the non-response rate. Only 12 studies have adequately described the non-response rate in the study^21,24,27,32,37,38,40,51,56,64,79,83^ (Supplementary Table 3). The mean NOS score assessing the impact of diabetes among TB treatment outcome was seven (of a possible nine points), suggesting that the high quality of the studies was included in the meta-analysis (Supplementary Table 6).

### A meta-analysis of the prevalence of diabetes among TB patients

**Figure 1:**
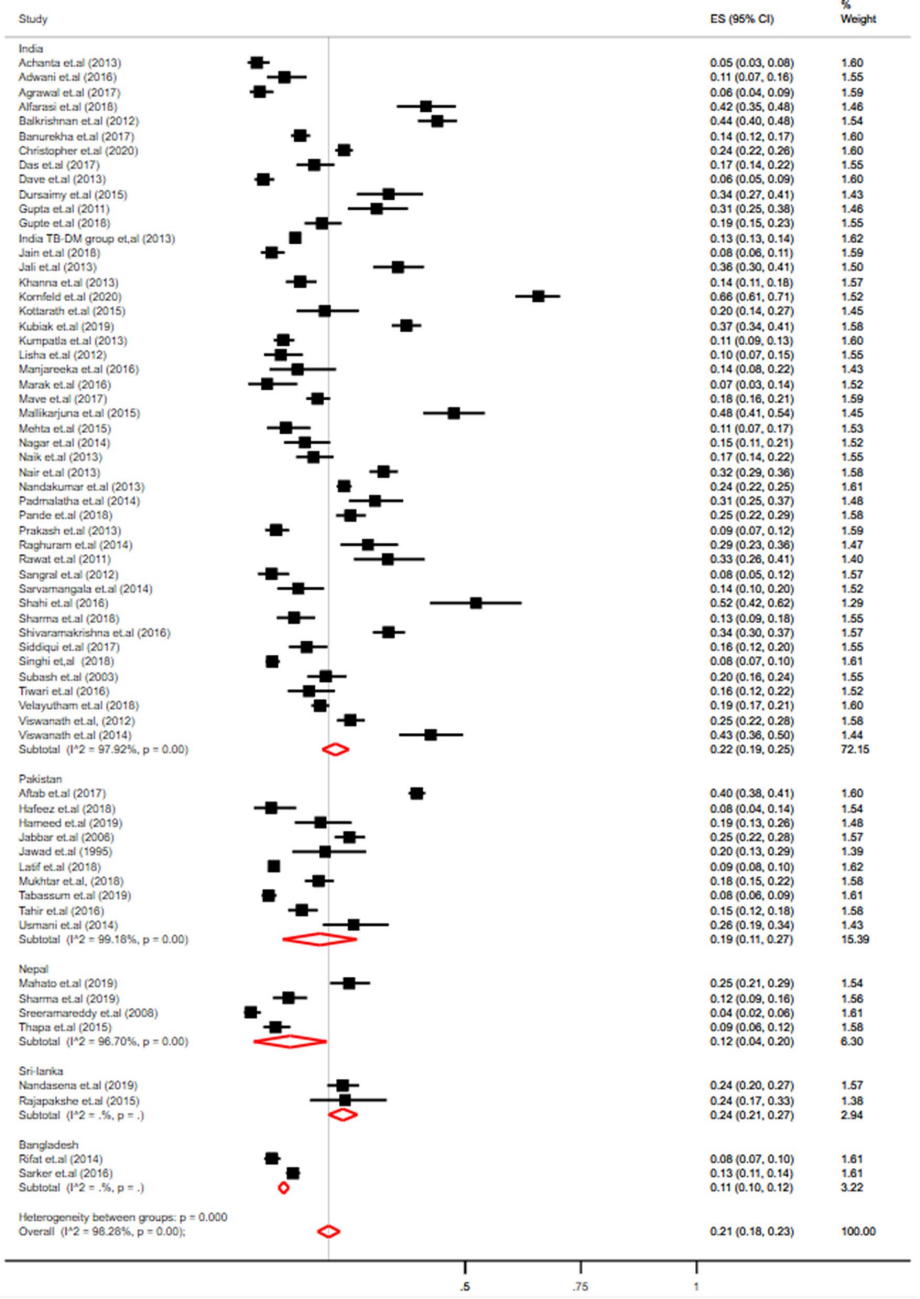
Forest plot for the pooled prevalence of diabetes among tuberculosis patients in South Asia.

A meta-analysis of 65 studies using the random-effect model showed that the overall pooled prevalence of diabetes among TB patients in South Asian countries was 21% (95% CI 18, 23) with-high heterogeneity (*I*^*2*^ = 98.28%, p-value < 0.001). The lowest prevalence of diabetes among patients with TB was 4% (95% CI 2.0, 6.0) which was reported from a study in Nepal^80^ and the highest was 66% (95% CI 61.0, 71.0) reported by a study in India^32^ (Fig. 1). In a sensitivity analysis, including only the studies with low risk of bias, there was no difference in the pooled prevalence of diabetes in tuberculosis patients (23%; 95% CI 19.0, 27.0, *I*^*2*^ = 98.85) (Supplementary Fig. 3). A visual analysis of the funnel plot showed some evidence of publication bias which was further confirmed by Egger’s test (p-value < 0.001).

Findings from subgroup analysis showed that there is a wide difference between the pooled prevalence across the countries; the lowest being 11% (95% CI 10.0, 12.0) in Bangladesh to the highest 24% (95% CI 21.0, 27.0) in Sri-Lanka. Similarly, a large variation in the diabetes prevalence was also seen within countries, ranging from 5% (95% CI 3.0, 8.0)^13^ to 66% (95% CI 61.0, 71.0)^32^ in India, 8%, (95% CI 4.0, 14.0)^76^ to 40% (95% CI 38.0, 41.0)^68^ in Pakistan, 4% (95% CI 2.0, 6.0)^80^ to 25% (95% CI 21.0, 29.0)^78^ in Nepal and 8% (95% CI 7.0, 10.0)^82^ to 13% (95% CI 11.0, 14.0)^83^ in Bangladesh (Table 1). Furthermore, a small reduction in the pooled prevalence of diabetes among the TB population was observed in the pooled analysis of studies limited to more than 300 participants only (19.0%, 95% CI 16.0, 22.0) and studies conducted within 2010–2015 (19.0%, 95% CI 16.0, 22.0). Additionally, the prevalence was higher in studies including only adults (21.0%, 95% CI 18.0, 23.0). Nevertheless, the subgroup analysis still showed considerable heterogeneity (> 90%) (Table 1). Meta-regression showed that the diabetes prevalence is associated with the period of data collection (*R*^2^ = 5.47%), diabetes diagnostic method (*R*^2^ = 9.96%) and the proportion of men in the sample (*R*^2^ = 10.74%). However, none of the covariates (countries, population, site, source of data collection, TB burden in the country) were found to be significant predictors to the observed heterogeneity in the meta-regression (Supplementary Table 4).

**Table 1:** Subgroup analysis of the prevalence of diabetes in people with tuberculosis.

Overall	Number of studies	Prevalence (95% CI)	% *I*^2^	P_heterogenity_
Total	65	21% (18.0–23.0)	98.28	< 0.0001
**By period**				
Before 2010	6	22.0% (11.0–33.0)	97.87	< 0.0001
2010–2014	36	19.0% (16.0–22.0)	97.68	< 0.0001
2015 and onwards	23	23.0% (17.0–28.0)	98.81	< 0.0001
**By sample size**				
Less than 300	25	23.0% (18.0–27.0)	94.66	< 0.0001
300 or more	40	19.0% (16.0–22.0)	98.79	< 0.0001
**By TB burden in-country**				
Low	6	16.0% (8.0–24.0)	97.15	< 0.0001
High	59	21.0% (19.0–24.0)	98.33	< 0.0001
**By site**				
Hospital-based	61	21.0% (18.0–23.0)	98.30	< 0.0001
Population-based	3	17.0% (9.0–26.0)	–	–
Both	1	24.0% (22.0–25.0)	–	–
**By population**				
Adults	55	21.0% (18.0–23.0)	97.99	< 0.0001
Adults and children	10	19.0% (13.0–25.0)	98.72	< 0.0001
**By country**				
Bangladesh	2	11.0% (10.0–12.0)	–	–
India				
Southern Region	26	27% (22.0–31.0)	98.10	< 0.0001
Western Region	8	15% (10.0–21.0)	96.24	< 0.0001
Eastern Region	2	17% (13.0–20.0)	–	–
Northern Region	3	14% (12.0–17.0)	–	–
North-Eastern Region	5	21% (11.0–30.0)	96.68	< 0.0001
Multiple Regions	3	14% (10.0–19.0)	–	–
Total pooled (India)	47	22.0% (19.0–25.0)	97.92	< 0.0001
Nepal	4	12.0% (4.0–20.0)	96.70	< 0.0001
Pakistan	10	19.0% (11.0–27.0)	99.18	< 0.0001
Sri-Lanka	2	24.0% (21.0–27.0)	–	> 0.0001

### Impact of diabetes on TB treatment outcomes

A total of nine met the eligibility criteria out of the 168 initially identified studies. Studies were excluded if they were not in English language, were conducted among people with severe illness or conditions and were not from SAARC countries. However, only eight studies were included in the meta-analysis^23,41,48,53–55,86,87^, a single study that reported hazard ratio (HR) was not included in the pooled analysis. All of the included studies were from India and had cohort design; out of which six were retrospective^23,41,48,53,55,87^ and the remaining three were prospective cohort studies^54,55,86^. The sample size for the non-diabetic TB population ranged from 120 to 2127, whereas for diabetic TB patients, it was 12 to 667. Additionally, the proportion of male participants in studies ranged from 44.6 to 77.69% and the average age of the participants was between 35 and 49 years old. The detailed characteristics of the included studies can be found in the supplementary (Supplementary Table 5).

Of eight studies, one study reported culture conversion at 2/3 months^88^, three studies reported mortality, failure, and conversion to MDR-TB^41,55,89^, two reported mortality and failure^48,55^, one reported mortality in terms of HR^87^, one reported a conversion to MDR-TB^53^ and 1 study reported recurrence as the TB treatment outcome^54^ (Supplementary Table 6).

#### Mortality

**Figure 2:**
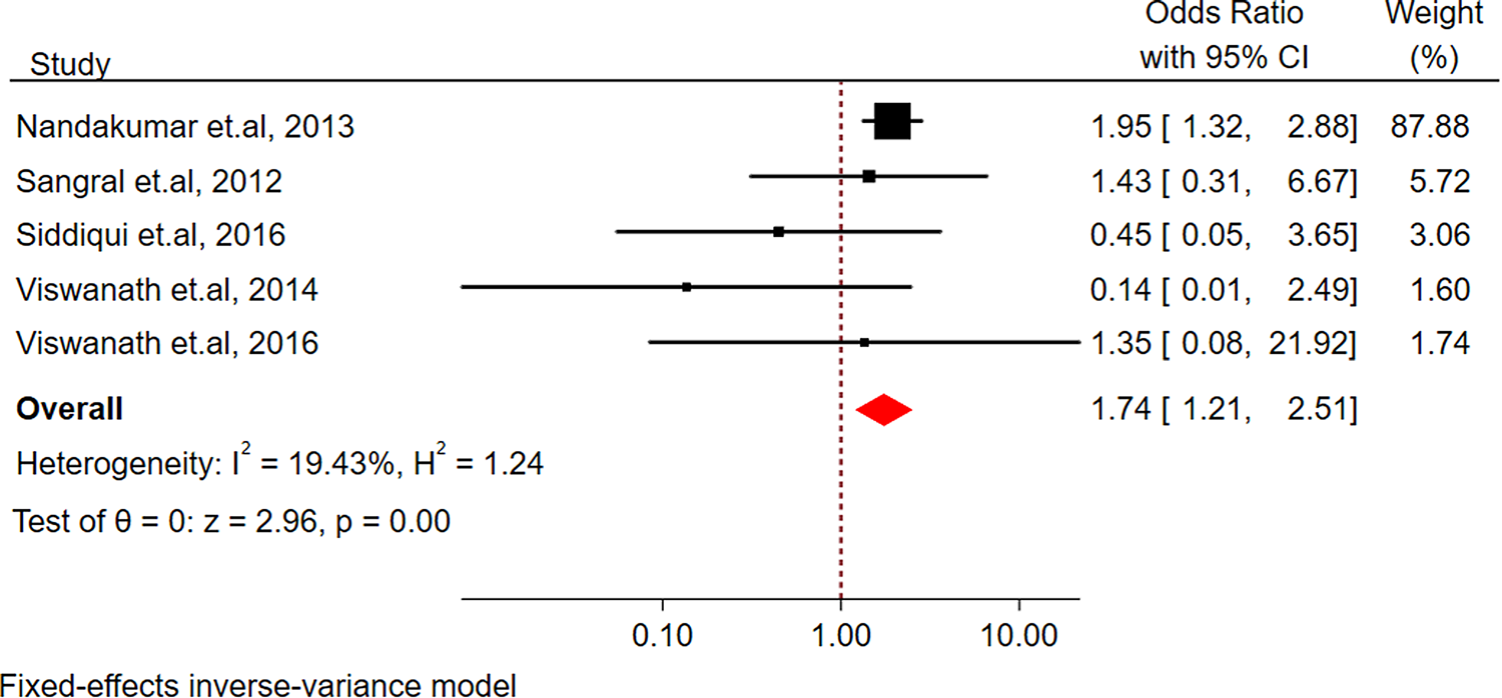
Forest plot for the association between diabetes and mortality among tuberculosis patients in South Asia.

The pooled estimates demonstrated that TB patients with diabetes had an approximately two-fold higher risk of mortality as compared with non-diabetic TB patients (Odds ratio (OR) = 1.74, 95% CI 1.21, 2.51; *I*^2^ 19.43%; low heterogeneity) as shown in Fig. 2. However, a single study, could not be pooled in the analysis as it reported only HR (1.30, 95% CI 0.16, 10.49), but still confirmed the higher risk of mortality as compared to non-diabetic TB patients^87^.

#### Treatment failure

**Figure 3:**
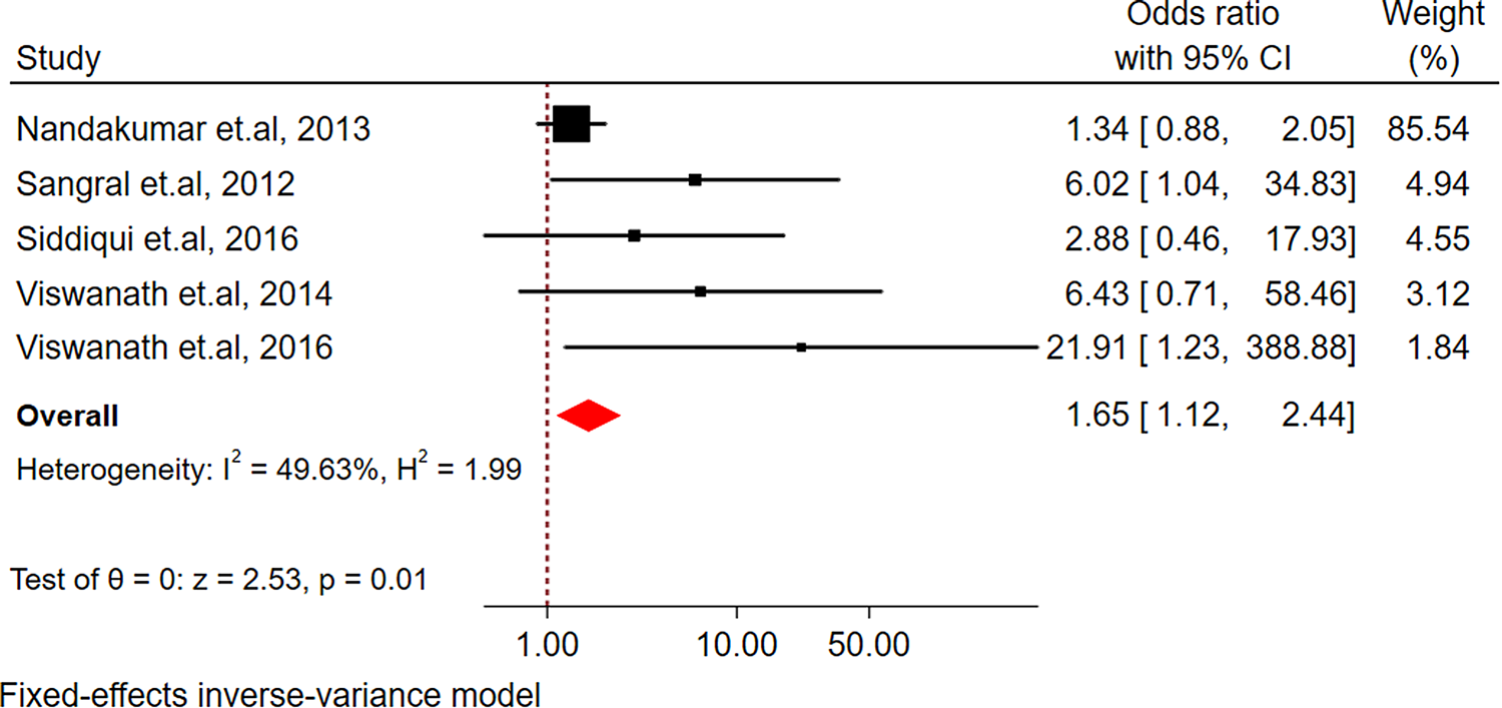
Forest plot for the association between diabetes and treatment failure among tuberculosis patients in South Asia.

The pooled result across all five studies reported a higher risk of treatment failure in TB participants with diabetes compared to non-diabetic TB participants (OR 1.65, 95% CI 1.12, 2.44) as shown in Fig. 3. There was moderate heterogeneity (*I*^2^ = 49.63%) across the pooled studies.

#### Multi drug-resistant tuberculosis

**Figure 4:**
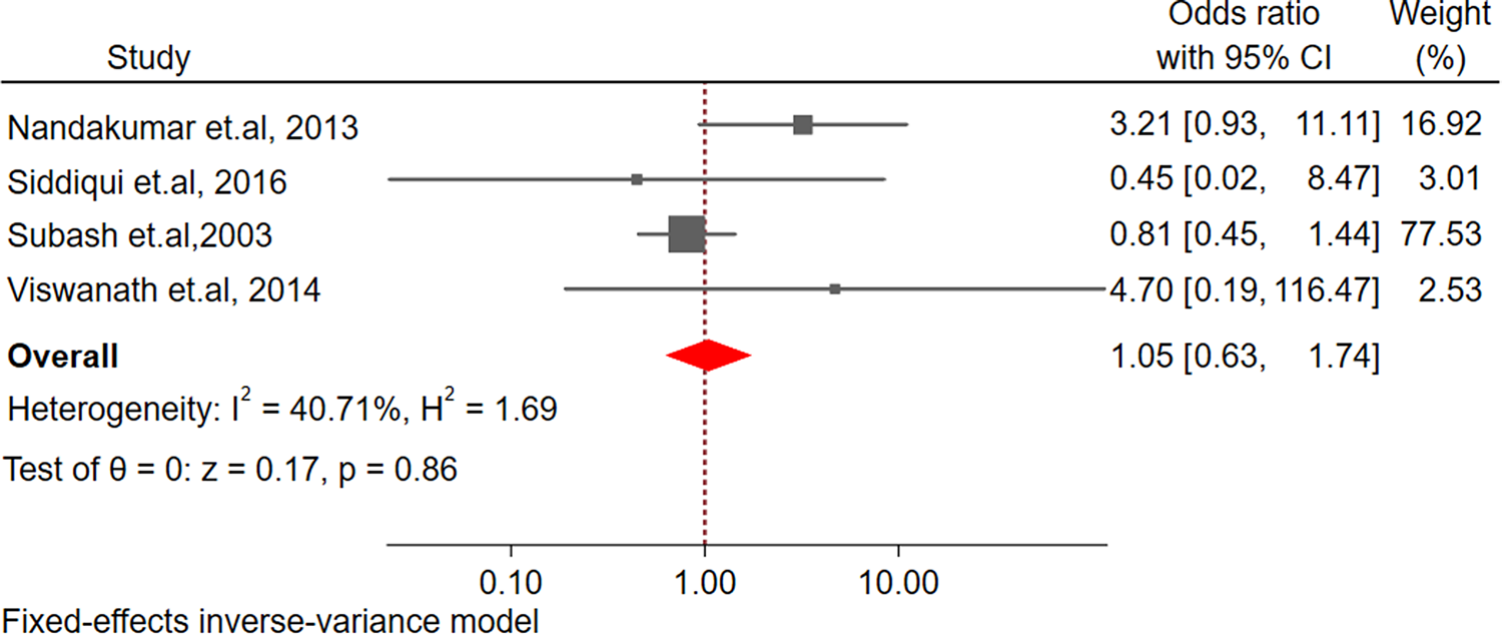
Forest plot for the association between diabetes and multidrug-resistant tuberculosis among tuberculosis patients in South Asia.

The pooled effect size demonstrated a nonsignificant difference in risk for MDR-TB between diabetic and non-diabetic TB participants (Fig. 4).

#### Culture conversion and recurrence

There was only one study reporting culture conversion as the treatment outcome which reported no significant association between diabetes and culture conversion (OR 0.32, 95% CI 0.10, 1.05)^88^. Additionally, a single study by Velayutham et al.^54^ reported the recurrence as the outcome. Compared with non-diabetic TB patients, diabetic TB patients had 0.53 lower odds (95% CI 0.32, 0.87) of recurrence after the TB treatment.

## Discussions

Our study found a higher prevalence of diabetes (21%, 95% CI 18.0, 23.0) among TB patients compared to Asia (17%; interquartile range 11.4, 25.8)^90^, and global prevalence, (15·4%, 95% CI 14.1, 16.6)^3^, and a marginally higher prevalence compared to a previous study from Southeast Asia (19%, 95% CI 16.2, 21.9)^3^. These variances could be elucidated by the fact that most of the Asians as compared to other populations of a known body mass index have higher visceral fat or adiposity, poor glycemic control, decreased pancreatic beta-cell mass and have reduced insulin secretion^91–94^.

There was high heterogeneity in the pooled estimates of diabetes prevalence among the TB patients across the studies (*I*^2^ = 98.28%, p-value < 0.001). In the subgroup analysis by the country, a wide variation in the diabetes prevalence in TB patients was found with the highest prevalence reported in Sri Lanka (24%) and the lowest prevalence reported in Bangladesh (11%). Furthermore, the prevalence of diabetes among TB patients also varied widely within countries itself from 5 to 66% in India, from 8 to 40% in Pakistan, from 4 to 25% in Nepal and from 8 to 13% in Bangladesh (Fig. 3). The variation in the prevalence of diabetes comorbidity among TB patients closely aligns with a higher burden of diabetes overall, and regional variation in diabetes prevalence: Southern states like Kerala and Tamil Nadu have a higher prevalence of diabetes compared to other states in India^95^ (Supplementary Fig. 4). Additionally, the prevalence of diabetes among the TB population was slightly higher in the sample size of less than 300 (23.0%, 95% CI 18.0, 27.0) compared to the sample size of more than 300 (19.0%, 95% CI 16.0, 22.0). Furthermore, the prevalence was higher in studies including only adults (21.0%, 95% CI 18.0, 23.0) and studies carried out in the high-risk countries of TB burden as classified by WHO (21.0%, 95% CI 19.0, 24.0) than the countries with a low risk of TB burden (16.0%, 95% CI 8.0, 24.0). In the meta-regression proportion of males in the study sample, diabetes diagnostic method and studies conducted in and after the year 2015 were found to explain the observed heterogeneity to some extent. The differences in dietary habits and diabetes screening methods may contribute to further variations in the prevalence of diabetes among TB patients within these countries^11,96,97^. These variances are also linked to overall variability in the caseloads of TB-diabetes co-morbidity, family history, and ethnic-mix of the population in South Asia. Despite the heterogeneity, these data suggest a higher prevalence of diabetes among TB patients in the South Asian population consistent with previous studies^3,98^ and warrants urgent attention from stakeholders.

### Impact of diabetes on tuberculosis treatment outcome

Data from this review showed that TB-diabetes patients have 1.74 (95% CI 1.21, 2.51) times higher odds of mortality compared to non-diabetic TB patients. This increased risk of mortality in TB diabetes comorbidity corroborates with previous systematic reviews^12,99^. These results depict a worrying picture of the effect of diabetes among TB patients especially among countries where the prevalence of diabetes is increasing rapidly. However, this finding was driven by only a single study in the meta-analysis and remaining four studies in the meta-analysis and a single study that could not be pooled in the meta-analysis showed no association. This is apparently due to absence of large longitudinal studies assessing impact of diabetes on TB treatment.

Additionally, TB patients with diabetes seemed to have a higher likelihood of treatment failure (OR = 1.65; 95% CI 1.12, 2.44). This finding is buttressed by previous studies where there was a higher failure rate in diabetic than non-diabetic TB patients^100–103^. Contrary to our results, Singla et al.^104^ and Khanna et al.^105^ reported no difference in TB treatment outcomes among diabetic and non-diabetic TB patients, which can be ascribed to small sample size and the retrospective design of these studies^105^.

We found no evidence that diabetes increases the risk of MDR-TB consistent with a previous study^103^. However, our study findings are contrary to results from another systematic review, which showed increased odds of MDR-TB among the TB- diabetes population^99,106–110^. However, the absence of association in our review might be due to the limited number of studies. Further, there was only one study that reported relapse and sputum conversion, however, the number of studies from other regions have reported increased odds of sputum conversion and relapse/recurrence^103,111,112^ among patients with TB-diabetes co-morbidity.

### Strength and limitations

The findings of this study need to be interpreted with caution due to the inherent limitations of the included studies as well as data availability for pooled analysis. We could not conduct subgroup analysis for various covariates that might be important for increased diabetes risk in TB patients such as age, education, and family income. Additionally, there is a possibility of differential misclassification of the outcome if blood glucose was examined before the initiation of TB treatment, as, TB infection can induce hyperglycemia that could be misdiagnosed as diabetes^113^. Various diagnostic techniques used for ascertaining diabetes in the TB population might have induced the risk of under or over-representation of diabetes patients among studies.

Also, a retrospective cohort study that had no data on glycemic control before the onset of TB made it arduous to ascertain the clinical manifestations of TB and temporal relationship (causal pathway) of glycemic control^16^. Other limitations include the lack of information on cause-specific deaths. This might have exaggerated the results since it cannot be assumed that increased deaths imply increased mortality due to TB; the deaths could be ascribed to any other comorbidities and factors inherent in the health system of a country.

A large number of studies included were from India, therefore the pooled estimates might not be generalizable to the whole South Asian region. Additionally, one study by Nandakumar et al.^41^ for assessing the impact of diabetes on tuberculosis treatment outcome contributed > 80% of the weight in our effect estimates which might have skewed our results.

Despite these limitations, to the best of our knowledge, this is the first systematic review and metanalysis to assess the prevalence of diabetes among TB patients and its impact on tuberculosis treatment in South Asia. The study was conducted adhering to the rigorous methodological protocol, designed according to the PRISMA guidelines for systematic reviews. The searches for eligible studies were conducted in electronic databases: Medline, Embase, and CINAHL which cover a wide range of peer-reviewed articles using broad search terms which ensured that relevant studies were unlikely to be missed. Additionally, references in the included studies and existing reviews were screened and forward citation tracking were done to identify relevant studies.

## Conclusion

In summary, the study found that a quarter of TB patients in South Asia also have diabetes comorbidity: 11% in Bangladesh to 24% in Sri-Lanka. These variations were seen not only across the countries but also within countries like India. Furthermore, the findings of this review show poorer treatment outcomes among diabetic TB patients compared to those without diabetes comorbidity; this was especially true for outcomes such as mortality and treatment failure. Considering the escalating burden of diabetes mellitus, especially in countries where the burden of TB still high, the provision of comprehensive screening programs might be beneficial for early diagnosis and treatment of diabetes among TB patients and vice-versa.

## Methods

### Search strategy and selection criteria

This systematic review and meta-analysis was done adhering to Preferred Reporting Items for Systematic Reviews and Meta-Analyses (PRISMA) guidelines^114,115^. Eligible studies reporting the prevalence of diabetes among TB patients were identified using a comprehensive search of peer-reviewed articles in PubMed, Embase, and CINAHL from January 1980 to 30 July 2020. The search strategy included all the commonly used terms for diabetes and TB as well as individual country names. For assessing the effect of diabetes on the TB treatment outcome, a separate search was conducted and search limited to articles published since 1980, as Rifampicin was included in the TB treatment regimen only since 1980^99^. Besides this, supplemental searches were conducted by examining the reference list of all the included studies and systematic reviews on this topic. The detailed search strategy for all the databases are included in the supplementary files (Supplementary Table 1).

### Inclusion and exclusion criteria

All relevant studies were screened for eligibility independently by two authors (SG, SM) and any discrepancies were resolved through discussion with the third author (NS). For inclusion, studies had to fulfill the following criteria: (1) Study population: Cross-sectional and other observational studies conducted in at least one of the South Asian Association for Regional Cooperation (SAARC) countries i.e. Afghanistan, Bangladesh, Bhutan, India, Maldives, Nepal, Pakistan in any age group or any gender. (2) Exposure: Diabetes diagnosed either by a physician or identified based on measured random plasma glucose, fasting plasma glucose, oral glucose tolerance tests according to WHO (World Health Organization) guidelines, HbA1c, anti-diabetic drugs, or self-reports (3). Comparison: For prevalence estimate of diabetes in TB patient those studies which were conducted among TB patients and reported the number of diabetic cases and TB patient population. For TB-treatment outcome, case–control and cohort studies assessing the difference in the TB treatment outcome between the TB-diabetes group and TB only patients. (4). Outcome: The included studies needed to have reported the prevalence of diabetes among patients with TB or provide data that allowed computation of the prevalence. For TB treatment outcome, studies needed to either report or provide data for assessing the impact of diabetes on at least one of the following five TB treatment outcomes:- treatment failure, mortality, relapse, and recurrence along with culture conversion (as a proxy of treatment outcome) and Multi-drug resistant TB (MDR-TB; as a long-term treatment outcome). During searches multiple articles using same dataset were also identified, in such cases the most comprehensive article that reported the outcomes considered in this review was considered for inclusion.

Exclusion criteria were: (1) Studies having a sample size less than 100 to avoid selection bias from small studies. For summarizing the treatment outcome, the studies were excluded if (1) used non-standard TB treatment regimens or different treatment regimens among TB patients with diabetes and without diabetes (2) were conducted among people with severe illness or conditions or in TB patients with proven drug resistance at the baseline as to have uniformity in treatments used and to make sure drug resistance doesn’t affect MDR-TB as treatment outcome.

### Data extraction and quality assessment

Two authors independently (SG and SM) extracted the data into a piloted data collection form adapted from Alebel et al.^9^ and Noubiap et al.^3^ with necessary modifications. Extracted data for assessing the prevalence of diabetes among TB patients include characteristics of studies, publication date, period of data collection, country included in the study, study design, sampling technique, sample size, gender and mean age of participants, diabetic cases among TB patients, method of data collection, a diagnostic method for TB and diabetes. Similarly, details were recorded regarding the assessment of TB treatment outcome, information on the type of outcome, specification of the outcome, wherever applicable, and available for assessing the impact of diabetes on TB treatment outcomes. The authors were contacted to provide additional information if needed. A modified version adapted from Alebel et al.^9^ of New castle Ottawa scales (NOS) was used to evaluate the quality of the included studies. The NOS scale assess risk of bias in three domains and assigns higher points for low risk of bias in each domain with maximum of nine points: (1) selection of study groups (four points); (2) comparability of groups (two points); and (3) ascertainment of outcomes (three points) . Studies with six points or higher were deemed to at of low risk of bias and consequently of high quality^9^.

### Statistical analysis

A high heterogeneity in prevalence estimates is expected when pooling the studies conducted in different populations^116^. However, the aim of this study is to summarize the prevalence of diabetes in TB patient in South Asia which can be then used to inform the likely prevalence of diabetes in TB patients in SAARC countries with similar population characteristics where no primary estimates are available^117^. The random-effect model was used to pool the prevalence of diabetes among TB patients, considering the likelihood of heterogeneity between studies which was estimated using the *I*^2^ statistic. Generally, *I*^2^ values greater than 70% suggest high heterogeneity^118^. The subgroup analysis was conducted according to the country, sample size (less than 300 or more than 300), study site (hospital-based/population-based/both), TB burden in the country (high/low), and by populations (adults/adults and children) for investigating the potential source of heterogeneity. The studies with only low risk of bias were pooled in a sensitivity analysis to assess the robustness of the findings. Furthermore, a funnel plot was generated to visually assess publication bias and was confirmed by the Eggers test. A p-value of less than 0.05 was considered statistically significant. The study-specific variance in prevalence was stabilized using the Freeman-Tukey double arcsine transformation for constructing the funnel plot^119^. Similarly, the random-effects meta-regression was conducted to investigate the potential sources of heterogeneity.

The treatment outcomes that were assessed were mortality, failure, development of MDR-TB, sputum conversion, and recurrence. The fixed-effect meta-analysis model was used for pooling the estimates of the impact of diabetes on TB treatment outcomes. The availability of only a few studies (less than five articles for each outcome) and the absence of significant heterogeneity were the reasons for selecting the fixed effect over the random effect model. All the analyses were performed in STATA. This systematic review was registered with the International Prospective Register of Systematic Reviews (PROSPERO), number CRD42020167896.

### Ethical approval and consent to participate

This article is based on previously conducted studies and does not contain any studies with human participants or animals performed by any of the authors.

## Supplementary Information

Supplementary Information
