# Supplementary material for: Diabetes among tuberculosis patients and its impact on tuberculosis treatment in South Asia: a systematic review and meta-analysis
Source: Sci Rep. 2021 Jan 22;11:2113. doi: 10.1038/s41598-021-81057-2 (PMC7822911; doi:10.1038/s41598-021-81057-2)
Supplement: Supplementary file 1 — Supplementary Information [file 41598_2021_81057_MOESM1_ESM.docx]

**Diabetes among tuberculosis patients and its impact on tuberculosis treatment in South Asia: A systematic review and meta-analysis**

Sanju Gautam, Nipun Shrestha, Shiva Raj Mishra, Sweta Mahato, Tuan P.A. Nguyen, Gabriele Berg-Beckhoff

**Supplementary Appendix**

**Tables**

1. Detailed search strategy for assessing the pooled prevalence of diabetes among TB patients in the South Asia Region
2. Detailed Characteristics of the included studies assessing the prevalence of diabetes among TB patients.
3. Bias Analysis of the studies included in the meta-analysis for assessing the prevalence of diabetes among TB patients
4. Meta-regression analysis for the variation of the prevalence of diabetes among TB patients
5. Detailed Characteristics of the included studies assessing the impact of diabetes on TB treatment outcome in South Asia
6. Bias Analysis of the studies included in the meta-analysis for assessing the impact of diabetes on tuberculosis treatment outcomes

**Figures**

1. Flowchart of summary of the search for articles related to the prevalence of diabetes and its impact on tuberculosis treatment outcome in the South Asia region
2. Funnel plot of the pooled prevalence of diabetes among TB patients in South Asia
3. Pooled prevalence of diabetes among TB patients in South Asia including low risk of bias studies
4. Pooled prevalence of diabetes among TB patients in India by region

**Supplementary Table 1: Detailed search strategy for assessing the pooled prevalence of diabetes among TB patients in the South Asia Region**

1. PubMed

| **SN** | **Query** | **Items found** |
| --- | --- | --- |
| 1 | ((Tuberculosis [MeSH Terms]) OR Tuberculosis) OR TB | 287390 |
| 2 | ((((Diabetes Mellitus) OR Diabetes Mellitus [MeSH Terms]) OR diab*) OR glucose*) OR *glucose | 1168057 |
| 3 | Search (((((((((Asia) OR India) OR Nepal) OR Pakistan) OR Bhutan) OR Maldives) OR Sri lanka) OR Bangladesh OR Afghanistan)) OR ((South Asian Association for Regional Cooperation) OR SAARC*) | 1408130 |
| 4 | (((((Tuberculosis[MeSH Terms]) OR Tuberculosis) OR TB)) AND (((((Diabetes Mellitus) OR Diabetes Mellitus[MeSH Terms]) OR diab*) OR glucose*) OR *glucose)) AND ((((((((((Asia) OR India) OR Nepal) OR Pakistan) OR Bhutan) OR Maldives) OR Sri lanka) OR Bangladesh) OR Afghanistan) OR ((South Asian Association for Regional Cooperation) OR SAARC*)) | 1292 |
| 5 | Search: (((Tuberculosis[MeSH Terms]) OR ((Tuberculosis) OR (TB))) AND (((diabetes mellitus[MeSH Terms]) OR ((diabetes mellitus) OR (diab*))) OR ((glucose*) OR (*glucose)))) AND (((((((((((Asia, western[MeSH Terms]) OR (Asia)) OR (India)) OR (Pakistan)) OR (Bangladesh)) OR (Bhutan)) OR (Maldives)) OR ((Sri Lanka) OR (Sri-lanka))) OR (Afghanistan)) OR (Nepal)) OR ((South Asian Association for Regional Cooperation) OR (SAARC))) Filters: Humans, English | 915 |

1. Embase

| SN | Query | Items found |
| --- | --- | --- |
| 1 | exp tuberculosis/ OR tuberculosis. af. OR TB.af | 385911 |
| 2 | exp diabetes mellitus/ OR diabetes mellitus.af. OR diab*.af OR *glucose/ OR glucose*.af. | 1884604 |
| 3 | (Asia or India or Nepal or Bangladesh or Pakistan or Bhutan or Sri Lanka or Maldives or Afghanistan or South Asian Association for regional cooperation or SAARC*).af. | 1413013 |
| 4 | 1 AND 2 AND 3 | 2587 |
| 5 | Limit 4 to human and English language | 2332 |

1. CINAHL

| SN | Query | Items found |
| --- | --- | --- |
| 1 | (MH "Tuberculosis+") OR tuberculosis OR TB | 29639 |
| 2 | (MH "Diabetes Mellitus+") OR diabetes mellitus OR diab* OR glucose* OR *glucose | 272493 |
| 3 | Asia OR India OR Nepal OR Bangladesh OR Sri Lanka OR Pakistan OR Bhutan OR Maldives OR Afghanistan OR South Asian Association for Regional Cooperation OR SAARC* | 77,801 |
| 4 | 1 AND 2 AND 3 | 94 |
| 5 | Limit 4 to human and English language | 48 |

### **For assessing the impact of diabetes on tuberculosis treatment outcome in the South Asia Region.**

1. PubMed

| SN | Query | Items found |
| --- | --- | --- |
| 1 | ((Tuberculosis [MeSH Terms]) OR Tuberculosis) OR TB | 287390 |
| 2 | ((((Diabetes Mellitus) OR Diabetes Mellitus [MeSH Terms]) OR diab*) OR glucose*) OR *glucose | 1168057 |
| 3 | Search (((((((((Asia) OR India) OR Nepal) OR Pakistan) OR Bhutan) OR Maldives) OR Sri lanka) OR Afghanistan) OR Bangladesh)) OR ((South Asian Association for Regional Cooperation) OR SAARC*) | 1408130 |
| 4 | (((((Tuberculosis[MeSH Terms]) OR Tuberculosis) OR TB)) AND (((((Diabetes Mellitus) OR Diabetes Mellitus[MeSH Terms]) OR diab*) OR glucose*) OR *glucose)) AND ((((((((((Asia) OR India) OR Nepal) OR Pakistan) OR Bhutan) OR Maldives) OR Sri lanka) OR Bangladesh)OR Afghanistan)) OR ((South Asian Association for Regional Cooperation) OR SAARC*)) | 1292 |
| 5 | Search ((treatment outcome [MeSH Terms]) OR "Treatment Outcome*") OR Treatment Outcome | 1344054 |
| 6 | Search (((((((("trial*") OR "Randomized Controlled Trial "[Publication Type]) OR randomized controlled trial[MeSH Terms]) OR Randomized Controlled Trials as Topic[MeSH Terms]) OR "Randomized Controlled Trials")) OR ((((cohort studies[MeSH Terms]) OR cohort studies) OR cohort analysis) OR cohort study))) OR ((Risk Factors[MeSH Terms]) OR Risk Factors) | 4,453,685 |
| 7 | 1 AND 2 AND 3 AND 4 AND 5 AND 6 | 147 |
| 8 | LIMIT 7 TO HUMANS AND ENGLISH | 133 |

1. Embase

| SN | Query | Items found |
| --- | --- | --- |
| 1 | exp tuberculosis/ OR tuberculosis. af. OR TB.af | 385911 |
| 2 | exp diabetes mellitus/ OR diabetes mellitus.af. OR diab*.af OR *glucose/ OR glucose*.af. | 1884604 |
| 3 | (Asia or India or Nepal or Bangladesh or Pakistan or Bhutan or Sri Lanka or Maldives or Afghanistan or South Asian Association for regional cooperation or SAARC*).af. | 1413013 |
| 4 | 1 AND 2 AND 3 | 2587 |
| 5 | exp treatment outcome/ or treatment outcome.af. or "treatment outcome*".af. | 1705532 |
| 6 | "Trial*".af. or exp randomized controlled trial/ or "randomized controlled trial".af. or exp cohort analysis/ or cohort analysis.af. or cohort study.af. or cohort studies.af. or exp risk factor/ or risk factor.af. | 4081713 |
| 7 | 4 AND 5 AND 6 | 132 |
| 8 | LIMIT 7 TO HUMAN AND ENGLISH | 129 |

1. CINAHL

| SN | Query | Items found |
| --- | --- | --- |
| 1 | (MH "Tuberculosis+") OR tuberculosis OR TB | 29639 |
| 2 | (MH "Diabetes Mellitus+") OR diabetes mellitus OR diab* OR glucose* OR *glucose | 272493 |
| 3 | Asia OR India OR Nepal OR Bangladesh OR Sri Lanka OR Pakistan OR Bhutan OR Maldives OR Afghanistan OR South Asian Association for Regional Cooperation OR SAARC* | 77,801 |
| 4 | 1 AND 2 AND 3 | 94 |
| 5 | (MH "Treatment Outcomes") OR “treatment outcome” OR treatment outcome | 376395 |
| 6 | "Trial*" OR (MH “randomized controlled trial”) OR "randomized controlled trial OR (MH "Prospective Studies+")OR cohort analysis OR cohort OR cohort study OR cohort studies OR (MH "Risk Factors+") OR “risk factors” OR “risk factor” | 473112 |
| 7 | 4 AND 5 AND 6 | 5 |
| 8 | LIMIT 7 TO HUMAN | 5 |

**Supplementary Figure 1:** **Flowchart of summary of the search for articles related to the prevalence of diabetes and its impact on tuberculosis treatment outcome in the South Asia region**

Records identified through database searching (for prevalence)

PubMed- 915

Embase-2332

CINAHL-48

Records identified through database searching (for treatment outcome)

PubMed- 110

Embase-53

CINAHL-5

569 duplicates removed

Records after duplicate removed

(n= 2894)

Records excluded based on title and abstract

(n=2700)

Records excluded based on full text for prevalence

- Review/Editorial/commentaries/ meeting correspondence/Conference abstract =33
- Study before 1980= 3
- No data available for calculation / not related outcome=10
- Full text not found= 6
- Coexistence of TB with other disease than diabetes/ no TB only group= 7
- Not south Asia=13
- Policy forum/framework/letter to editor/book, newspaper article/ annual report/guideline=11
- Less than 100 sample size=15
- Duplicate=5
- Wrong study design=3

Records assessed in full text

Prevalence: n= 166 + 5 articles added from reviewing the references

Treatment outcome: n=28

Records excluded based on full text for treatment outcome

- Review = 3
- No control group= 4
- Not related outcome/outcome not differentiated=5
- Not south Asia=3
- Patients with drug resistance at the baseline=3
- Cross sectional study=1

Studies included in the final analysis

Prevalence: n=65

Treatment outcome: n= 9

**Supplementary Table 2: Detailed Characteristics of the included studies assessing the prevalence of diabetes among TB patients.**

| The study, Publication year | Data collection time/ country | Type of study | Sampling | Hospital  Based (yes/No) | Mean/  median age with age group | No of TB Patients  Male/ female | Diagnosis of  TB | Population with diabetes | Diagnosis of diabetes |
| --- | --- | --- | --- | --- | --- | --- | --- | --- | --- |
| Achanta et.al, 2013^1^ | 2012  India | Cross-sectional  (prospectively) | Consecutive | Yes | NR | M: 236  F: 138 | Clinically, laboratory and X-Ray based | 19 | FPG |
| Adwani et.al,2016^2^ | 2016  India | Cross-sectional  (prospectively) | Consecutive | Yes | NR | M: 113  F: 114 | Clinically, laboratory-based | 25 | NR |
| Aftab et.al, 2017^3^ | 2014-15  Pakistan | Cross-sectional (prospectively) | Consecutive | Yes | 44.2 | M: 1127  F: 681 | Laboratory based | 1112 | HBA1c |
| Agrawal et.al, 2017^4^ | 2016  India | Cross-sectional (prospectively | Cluster random sampling | Yes | 33.1 | NR | Laboratory and X-ray | 20 | RPG |
| Alfarasi et.al, 2018^5^ | 2013-17  India | Cohort  (prospectively) | Consecutive | Yes | 48 | M: 164  F: 79 | Tuberculosis  patients | 101 | FPG, RPG or using antidiabetic |
| Balkrishnan, et.al, 2012^6^ | 2011  India | Cross-sectional (prospectively | Cluster sampling method | All the TB units of Kerala | 46 | M: 420  F:132 | Clinically, laboratory and X-Ray based | 243 | Self-report and HBA1C |
| Banurekha et.al, 2017^7^ | 2015-16  India | Cohort (retrospectively) | Consecutive | Yes | NR | NR | Treatment cards, registered patients | 163 | Treatment cards |
| Christopher et.al,2020^8^ | 2001-11  India | Cohort (retrospectively) | Consecutive | Yes | NR | M: 1405  F: 574 | Medical Records,  X-ray based | 472 | FPG or postprandial, records |
| Das et.al,2017^9^ | 2014-16  India | Cohort (prospectively) | Consecutive | Yes |  | M: 256  F: 94 | Registered patients | 61 | FPG, RPG, Antidiabetic medications |
| Dave et.al, 2013^10^ | 2012  India | Cohort (prospectively) | Consecutive | Yes | 35 | M: 371  F: 185 | Laboratory based | 36 | FPG |
| Duraisamy et.al, 2015^11^ | 2009-10  India | Cohort  (retrospectively) | NR | Population based | 45 | M: 139  F: 40 | Laboratory based | 60 | HBA1C |

| Gupta et.al, 2011^12^ | 2005-06  India | Cohort  (retrospectively) | Consecutive | Yes | NR | M: 163  F:44 | Clinically based, laboratory-based | 64 | NR |
| --- | --- | --- | --- | --- | --- | --- | --- | --- | --- |
| Gupte et.al, 2018^13^ | 2013  India | Cohort (prospectively) | Consecutive | Yes | 31 | M: 246  F: 146 | Smear microscopy, XPert® MTB/R | 75 | Self-report (taking antidiabetic) or HBA1c |
| Hafeez et.al,2018^14^ | 2016-17  Pakistan | Cross-sectional  (prospectively) | consecutive | Yes | 46.14 | M: 119  F: 40 | Clinically, laboratory and X-Ray based | 13 | FPG |
| Hameed et.al ,2019^15^ | 2018-19  Pakistan | Cross-sectional (prospectively) | Consecutive | Yes | NR | M: 92  F: 78 | Acid Fast Bacilli (AFB) smear | 32 | FPG and RPG |
| India Tb -DM group et.al, 2013^16^ | 2011-12  India | Cross-sectional (prospectively) | Consecutive | Yes | NR | NR | Lab based | 1084 | FPG |
| Jabbar et.al, 2006^17^ | 1992-96  Pakistan | Cross-sectional (Retrospectively) | Consecutive | Yes | 46.1 | M: 415 F: 276 | Clinically based, Laboratory-based, X-ray-based | 173 | OGTT, Taking antidiabetics |
| Jain et.al, 2018^18^ | 2013-16  India | Cohort (Retrospective and prospective) | Consecutive | Yes | 38 | M: 127  F:347 | Clinically based, Laboratory-based, X-ray-based | 39 | RPG |
| Jali et.al,2013^19^ | 2012  India | Cross-sectional (prospectively) | Consecutive | Yes | 50.7 | M:197  F:110 | Registered Tuberculosis patients | 109 | FPG |
| Jawad et.al, 1995^20^ | NR  Pakistan | Cross-sectional (prospectively) | Consecutive | Yes | 39.3 | M: 63  F: 43 | Laboratory and x-ray based | 21 | FPG, OGTT |
| Khanna et.al, 2013^21^ | 2012  India | Cross-sectional (Retrospective) | Consecutive | Yes | 32 | M: 226  F: 232 | NR | 66 | FPG, Taking antidiabetics |
| Kornfeld et.al, 2020^22^ | 2014-18  India | cohort (prospectively) | Consecutive | Yes | NR | NR | Laboratory-based | 256 | FPG, OGTT, HBA1c |
| Kottarath et.al, 2015^23^ | 2014-15  India | Cross-sectional (prospectively) | Consecutive | Yes | 45.39 | M: 105  F: 42 | Tuberculosis patients | 29 | FPG |
| Kubiak et.al, 2019^24^ | 2014-18  India | Cross-sectional (prospectively) | Consecutive | Yes | 44.9 | M: 729  F: 190 | Laboratory-based | 343 | Taking antidiabetics, RPG |
| Kumpatla et.al, 2013^25^ | 2010-11  India | Cross-sectional (prospectively) | Random | Yes | 50.3 | M: 528  F: 251 | Clinically based, Laboratory-based, X-ray-based | 84 | FPG, OGTT |
| Latif et.al, 2018^26^ | 2010-14  Pakistan | Cross-sectional (retrospectively) | Consecutive | Yes | 29 | M: 2970  F: 2841 | Tuberculosis register | 509 | FPG |
| Lisha et.al, 2012^27^ | 2008-10  India | Cohort (Retrospectively) | Consecutive | Yes | 47 | M: 182  F: 42 | Laboratory-based, X-ray-based | 23 | RPG |
| Mahato et.al, 2019^28^ | 2018-19  Nepal | Case-control (Prospectively) | Consecutive | Yes | NR | M: 253  F: 155 | Tuberculosis treatment centers | 102 | Plasma glucose examination |
| Marak et.al, 2016^29^ | 2013-14  India | Cross-sectional  (prospectively) | Consecutive | Yes | NR | M: 70  F: 40 | Registered Tuberculosis patients | 8 | RPG and FPG |
| Manjareeka et.al,2016^30^ | 2014  India | Cross-sectional  (prospectively) | Consecutive | Yes | 46.7 | M:76  F:25 | Laboratory-based | 14 | FPG |
| Mave et.al, 2017^31^ | 2013-17  India | Cross-sectional (prospectively) | Consecutive | Yes | 32 | M: 589  F: 301 | Clinically based, Laboratory-based | 162 | RPG, HbA1c |
| Mallikarjuna et.al, 2015^32^ | 2013-15  India | Cross-sectional (prospectively) | Consecutive | Yes | NR | NR | Clinically based, Laboratory-based | 109 | Antidiabetic medication, FPG |
| Mehta et.al,  2015^33^ | 2012-13  India | Cross sectional (prospectively | Consecutive | Yes | 37.4 | M: 151  F: 43 | Clinically based, Laboratory-based | 22 | Self-report |
| Mukhtar et.al, 2018^34^ | 2013-14  Pakistan | Cohort (Prospectively) | Consecutive | Yes | 50.8 | M: 312  F: 302 | Clinically based, Laboratory-based, X-ray-based | 113 | RPG, FPG, Self-report |
| Nagar et.al, 2015 ^35^ | 2013-14  India | Cross-sectional (prospectively) | Consecutive | Yes | NR | M: 118  F: 102 | standard diagnostic criteria  of RNTCP. | 34 | Standard diagnostic criteria of the American Diabetes  Association |
| Naik et.al, 2013^36^ | 2012  India | Cross-sectional (prospectively) | Consecutive | Yes | 40 | M: 246  F: 116 | Tuberculosis patients (register) | 62 | FPG, RPG |
| Nair et.al, 2013^37^ | 2012  India | Cross sectional (prospectively) | Consecutive | Yes | 47.6 | M: 670  F: 250 | Laboratory based | 298 | FPG |
| Nandakumar et.al, 2013^38^ | 2010-11  India | Cohort (retrospectively) | Consecutive | Hospital and population based | 46 | M: 1896  F: 898 | Laboratory-based | 667 | FPG, OGTT |
| Nandasena et.al, 2019^39^ | 2013  Sri-Lanka | Cross-sectional  (retrospectively) | Consecutive | Yes | NR | NR | Tuberculosis patients | 145 | Records |
| Padmalatha et.al, 2014^40^ | 2014  India | Cross-sectional (prospectively) | Consecutive | Yes | 41.5 (male)  33.4 (females | M: 170  F: 82 | Tuberculosis patients | 77 | FPG and taking antidiabetics |
| Pande et.al, 2018^41^ | 2015-16  India | Cohort (retrospectively) | Consecutive | Yes | NR | M: 517  F: 219 | Clinically based, Laboratory-based, | 184 | FPG, RPG |
| Prakash et.al,2013^42^ | 2012  India | Cross-sectional (prospectively) | Consecutive | Yes | 35 | M: 316  F: 194 | Clinically based, Laboratory-based, X-ray-based | 47 | FPG, Taking antidiabetics |
| Raghuram et.al, 2014^43^ | NR  India | Cross-sectional (prospectively) | Random | Yes | M-44.9  and Fe-36.2 | M: 172  F: 45 | NR | 63 | FPG, Taking antidiabetics |
| Rajapakshe et.al, 2015^44^ | 2013-14  Sri-Lanka | Cross sectional (prospectively | Consecutive | Yes | 51 | M: 81  F: 31 | NR | 27 | FPG |
| Rawat et.al, 2011^45^ | 2007-08  India | Cohort (prospectively) | Consecutive | Yes | 53.3 | M: 99  F: 57 | Clinically based, Laboratory-based, X-ray-based | 52 | FPG |
| Rifat et.al, 2014^46^ | 2012-13  Bangladesh | Case-control (Prospectively) | Consecutive | Yes | 36.9 | M: 609  F: 391 | Clinically based, Laboratory-based | 83 | Self-reported |
| Sangral et.al, 2012^47^ | 2009-10  India | Cross sectional  (prospectively) | Consecutive | Population based | Female:37.12  Male:43.1 | M: 183  F: 97 | Registered patients | 23 (15 male and 8 females | RPG, OGTT |
| Sarker et.al, 2016^48^ | 2013-14  Bangladesh | Cross-sectional (prospectively) | NR | Population based | 39.9 | M: 1170  F: 740 | Clinically based, Laboratory-based | 245 | OGTT, RPG |
| Sarvamangala et.al, 2014^49^ | 2011  India | Cross-sectional (prospectively) | Convenient sampling | Yes | 46.4 | NR | Laboratory-based | 28 | Records, blood examination  as per ADA criteria |
| Shahi et.al, 2016^50^ | 2013-15  India | Cross-sectional (prospectively) | Consecutive sampling | Yes | 51.2 | NR | Clinically based, Laboratory-based | 55 | FPG and postprandial, HbA1c |
| Sharma et.al, 2018^51^ | NR  India | Cross-sectional  (prospectively) | Random sampling to select study sites and consecutive to participants | Yes | 34.2 | M: 169  F: 106 | Registered Tuberculosis patients | 36 | Self-report, antidiabetics |
| Sharma et.al, 2019^52^ | 2018-19  Nepal | Cross-sectional  (prospectively) | Random sampling to select study sites and consecutive to participants | Yes | 41.5 | M: 224  F: 96 | TB patients on treatment | 38 | FPG and OGTT and antidiabetic treatment |
| Shivaramakrishna et.al ,2016^53^ | 2014  Nepal | Cross-sectional  (prospectively) | Random sampling to select study sites | Yes | 48.8 (M)  40.2(F) | M: 516  F: 212 | TB patients on treatment | 244 | FPG, Antidiabetic treatment |
| Siddiqui et.al, 2017^54^ | 2014  India | Cohort (prospectively) | Consecutive | Yes | 44 | M: 175  F: 141 | Clinically based, Laboratory-based | 50 | FPG, OGTT |
| Singhi et.al, 2018^55^ | 2016-17  India | Cohort (retrospectively) | Consecutive | Yes | NR | NR | Registered TB patients | 109 | NR |
| Sreeramareddy et.al, 2008^56^ | 2003-06  Nepal | Cross-sectional (retrospectively) | Consecutive | Yes | EPTB patients (29.5 years.  PTB patients (47.5 years) | M: 289  F: 185 | Registered TB patients | 18 | Records |
| Subash et.al, 2003^57^ | 1997-99  India | Cohort (retrospectively) | NR | Yes | 51 | M: 277  F: 84 | Clinically based, Laboratory-based | 72 | FPG, Taking antidiabetics |
| Tabassum et.al,2019^58^ | 2017  Pakistan | Cross-sectional (prospectively) | Convenient | Yes | NR | M:529  F: 591 | Registered patients | 85 | Self-report |
| Tahir et.al, 2016^59^ | 2014-15  Pakistan | Cross-sectional (prospectively) | NR | Yes | 38 | M: 340  F: 160 | Clinically based, Laboratory-based, X-ray-based | 74 | FPG, HbA1c |
| Thapa et.al,2015^60^ | 2013  Nepal | Cross-sectional (prospectively) | Purposive sampling | yes | 31.4 | M: 199  F: 208 | TB patients on treatment | 37 | RPG, Antidiabetic medication, record |
| Tiwari et.al,2016^61^ | 2015  India | Cross-sectional (prospectively) | Consecutive | Yes | NR | Total: 220 | Registered patients | 36 | RPG from Hospital records |
| Usmani et.al, 2014^62^ | 2011  Pakistan | Cross-sectional (prospectively) | Systematic sampling (random) | Yes | NR | M: 97  F: 61 | Clinically based, Laboratory-based, X-ray-based | 41 | FPG |
| Velayutham et.al, 2018^63^ | 2013-14  India | Cohort (prospectively) | Consecutive | Yes | 41 | M: 1125  F: 440 | Clinically based, Laboratory-based | 293 | FPG |
| Viswanath et.al, 2012^64^ | 2011  India | Cross-sectional (prospectively) | Cluster random sampling | Yes | 41.1 | M: 570  F: 257 | Clinically based, Laboratory-based, X-ray-based | 209 | OGTT, HbA1c, FPG |
| Viswanath et.al, 2014^65^ | 2011-12  India | Cohort (prospective) | Random for choosing TB units, consecutive for sample | Yes | TB-diabetes 50; TB :48 | M: 159  F: 50 | Registered TB patients | 89 | OGTT, |

#NR: Not Reported

FPG: Fasting plasma glucose

RPG: Random plasma glucose

#2H-OGTT: 2h Oral Glucose Tolerance Test

#EPTB: Extra Pulmonary Tuberculosis

HBA1C: glycosylated hemoglobin

**Supplementary Table 3: Bias Analysis of the studies included in the meta-analysis for assessing the prevalence of diabetes among TB patients**

| Author | Sample selection | | | | | Comparability | Outcome | | Total Quality Score |
| --- | --- | --- | --- | --- | --- | --- | --- | --- | --- |
|  | **Representativeness of the sample** | **Sample size** | **Non-respondent** | **Assessment of the Tuberculosis** | **Ascertainment of the exposure (risk factor)** |  | **Assessment of the outcome** | **Statistical test** |  |
| Achanta et.al ,2013 | A* | B | C | A* | A* | B | A** | B | 5 |
| Adwani et.al,2016 | B* | B | C | A* | B | B | D | B | 2 |
| Aftab et.al,2017 | B* | B | B | A* | A* | A** | A** | A* | 8 |
| Agrawal et. al, 2017 | A* | A* | A* | A* | A* | B | A** | A* | 8 |
| Alfarasi et.al,2018 | B* | B | C | A* | A* | A** | A** | A* | 8 |
| Balakrishnan et.al, 2012 | A* | A* | C | A* | B | A** | A** | A* | 8 |
| Banu Rekha et.al, 2017 | B* | B | C | A* | A* | B | b** | B | 5 |
| Christopher et.al,2020 | A* | B | C | B* | B | A** | B** | A* | 7 |
| Das et.al,2017 | B* | B | C | A* | B | B | A** | B | 4 |
| Dave et.al,2013 | A* | B | A* | A* | B | B | A** | B | 5 |
| Duraisamy et. al,2014 | C | B | C | A* | B | A** | A** | A* | 6 |
| Gupta et,al, 2011 | B* | B | C | A* | B | B | D | B | 2 |
| Gupte et.al,2018 | B* | B | A* | A* | B | A** | A** | A* | 8 |
| Hafeez et.al,2018 | B* | A* | C | A* | B | B | A** | B | 5 |
| Hameed et. al, 2019 | B* | B | C | A* | A* | B | A** | B | 5 |
| India Tb -DM group, 2013 | B* | B | C | A* | A* | B | A** | B | 5 |
| Jabbar et.al, 2006 | B* | B | C | A* | B | B | A** | B | 4 |
| Jain et.al, 2018 | A* | B | C | A* | A* | B | A** | B | 5 |
| Jali et.al, 2013 | A* | B | C | A* | A* | B | A** | B | 5 |
| Jawad et, al.1995 | B* | B | C | A* | B | B | A** | B | 4 |
| Khanna et.al, 2013 | B* | B | C | A* | B | B | A** | B | 4 |
| Kornfeld et.al, 2016 | B* | B | A* | A* | A* | B | A** | B | 6 |
| Kottarath et.al,2015 | A* | b | C | B* | B | A** | A** | A* | 7 |
| Kubiak et.al, 2019 | B* | B | C | A* | B | A** | A** | A* | 7 |
| Kumpatla et.al, 2013 | A* | B | C | A* | A* | B | A** | B | 5 |
| Latif et.al, 2018 | A* | B | C | A* | A* | B | A** | A* | 6 |
| Lisha et.al, 2012 | A* | A* | C | A* | A* | A** | A** | A* | 9 |
| Mahato et.al, 2019 | A* | A* | C | B* | A* | A** | A** | A* | 9 |
| Marak et.al, 2016 | B* | A* | A* | B* | B | B | A** | B | 6 |
| Manjareeka et.al, 2016 | B* | A* | A* | A* | B | B | A** | B | 6 |
| Mave et.al et.al,2017 | B* | B | C | A* | B | A** | A** | A* | 7 |
| Mallikarjuna et.al, 2015 | B* | B | C | A* | B | B | A** | B | 4 |
| Mehta et.al, 2015 | B* | B | C | A* | B | B | C* | B | 3 |
| Mukhtar et.al, 2018 | A* | A* | C | A* | B | A** | A** | A* | 8 |
| Nagar et.al, 2014 | B* | B | A* | A* | A* | B | A** | B | 6 |
| Naik et.al, 2013 | A* | B | C | A* | B | B | A** | B | 4 |
| Nair et.al, 2013 | B* | B | C | A* | A* | A** | A** | A* | 8 |
| Nandakumar et.al, 2013 | A* | A* | C | A* | A* | A** | A** | A* | 9 |
| Nandasena et.al, | B* | B | C | B* | B | B | B** | B | 4 |
| Padmalatha et.al,2014 | B* | B | C | B* | A* | B | A** | B | 5 |
| Pande et.al, 2018 | A* | B | C | A* | A* | A** | A** | A* | 8 |
| Prakash et.al, 2013 | A* | B | C | A* | A* | B | A** | B | 5 |
| Raghuram et.al, 2014 | A* | B | C | B* | A* | B | A** | A* | 6 |
| Rajapakshe et.al, 2015 | A* | B | C | B* | B | B | A** | B | 4 |
| Rawat et.al, 2008 | B* | B | C | A* | B | B | A** | B | 4 |
| Rifat et.al, 2014 | B* | A* | C | A* | A* | A** | C* | A* | 8 |
| Sangral et.al, 2012 | A* | B | C | B* | A* | B | A** | B | 5 |
| Sarker et.al, 2016 | B* | A* | A* | A* | A* | B | A** | A* | 8 |
| Sarvamangala et.al, 2014 | B* | B | C | A* | A* | B | A** | B | 5 |
| Shahi et.al,2016 | B* | B | C | A* | B | B | A** | B | 4 |
| Sharma et.al, 2018 | A* | A* | A* | B* | B | B | C* | A* | 6 |
| Sharma et.al,2019 | A* | A* | C | B* | A* | B | A** | B | 6 |
| Shivaramakrishna et,2016 | A* | A* | A* | B* | A* | B | A** | B | 7 |
| Siddiqui et.al, 2017 | B* | A* | A* | A* | A* | A** | A** | A* | 10 |
| Singhi et.al, 2018 | A* | B | C | B* | B | B | D | B | 2 |
| Sreeramareddy et.al, 2008 | B* | B | C | B* | B | A** | B** | A* | 7 |
| Subash et.al, 2003 | C | A* | C | A* | B | B | B** | B | 4 |
| Tabassum et.al,2019 | B* | B | C | B* | B | B | C* | B | 3 |
| Tahir et.al, 2016 | C | B | C | A* | A* | B | A** | A* | 5 |
| Thapa et.al, 2015 | B* | A* | C | B* | A* | B | A** | B | 6 |
| Tiwari et.al,2016 | B* | B | C | B* | A* | B | B** | B | 5 |
| Usmani et.al,2014 | A* | A* | C | A* | A* | B | A** | B | 6 |
| Velayutham et.al,2018 | B* | A* | C | A* | A* | A** | A** | A* | 9 |
| Viswanath et.al,2012 | A* | A* | A* | A* | A* | B | A** | A* | 8 |
| Viswanath et.al, 2014 | A* | A* | C | B* | A* | A** | A** | A* | 9 |

**Supplementary Figure 2: Funnel plot of the pooled prevalence of diabetes among TB patients in South Asia**

**Supplementary Figure 3:** **Pooled prevalence of diabetes among TB patients in South Asia including low risk of bias studies**


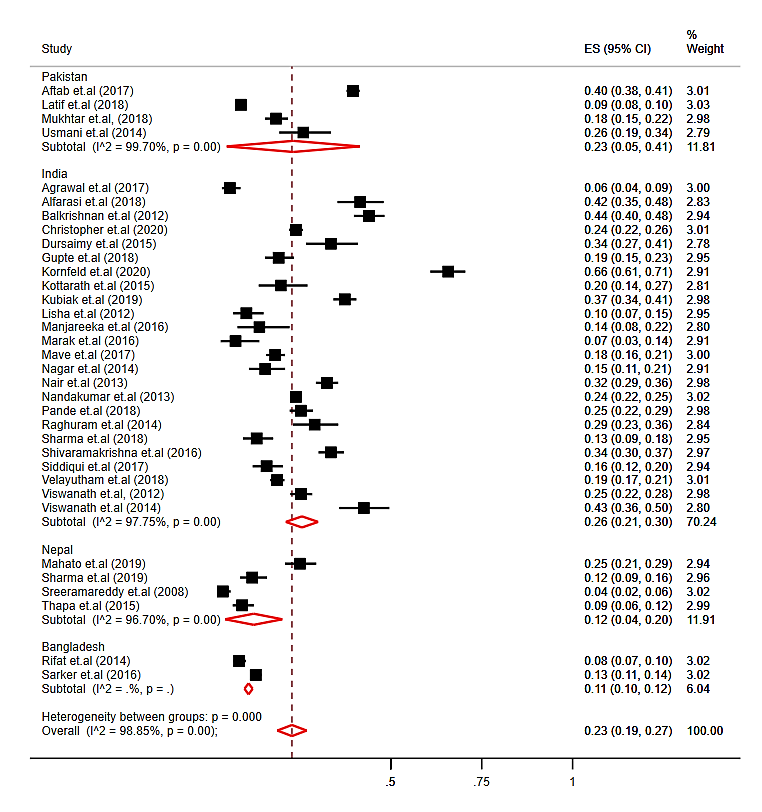


**Supplementary Table 4: Meta-regression analysis for the variation of the prevalence of diabetes among TB patients**

| **Variables**  **(reference)** | **Univariate analysis** | |
| --- | --- | --- |
|  | P-value | Coefficient(95%CI) |
| **% of Males***  (*missing values included) | 10.74% (*R^2^*%) | |
|  | 0.01 | 0.01 (0.01, 0.01) |
| **Period of data collection (Before 2015)** | 5.47% (*R^2^*%) |  |
| 2015 and afterwards | 0.03 | -0.10 (-0.19, -0.01) |
| **Sample size (less than 300)** |  |  |
| More than or equal to 300 | 0.23 | -0.05 ( -0.12, 0.03) |
| **Country (Bangladesh)** | 0.38 (overall P-value) 0.23% (*R^2^*%) | |
| India | 0.19 | 0.14 (-0.07, 0.36) |
| Pakistan | 0.29 | 0.12 (-0.11, 0.36) |
| Sri-Lanka | 0.24 | 0.18 (-0.12, 0.48) |
| Nepal | 0.91 | 0.15 (-0.24, 0.27) |
| **TB burden in the country (Low)** |  | |
| High | 0.33 | 0.06 (-0.65, 0.19) |
| **Site (Population based)** | 0.87 (overall P-value) | |
| Hospital based | 0.70 | 0.03 (-0.15, 0.21) |
| Both | 0.62 | 0.087 (-0.26, 0.44) |
| **Source of data collection (Directly from Participants part** | 0.59 (overall P-value) | |
| From medical files/databases | 0.54 | -0.03( -0.12, 0.06) |
| Both | 0.39 | -0.097 ( -0.32, 0.12) |
| **Populations (Adults)** |  | |
| Adults and children | 0.59 | -0.03 (-0.13, 0.07) |
| **Diabetes Diagnostic method (2h-OGTT and RPG, FPG)** | **0.041** (overall P-value) 9.96 (*R^2^*%) | |
| Antidiabetic treatment | 0.48 | -0.10 (-0.40, 0.19) |
| HBA1C, Antidiabetic treatment | 0.16 | 0.104 (-0.04, 0.25) |
| 2h-OGTT, FPG, HBA1C, Antidiabetic treatment | 0.04 | 0.330 (0.02, 0.64) |
| FPG, RPG, HBA1C, Antidiabetic treatment | 0.46 | -0.03(-0.12, 0.05) |
| Self-reported, Records | 0.08 | -0.13(-0.28, 0.01) |
| Not reported | 0.44 | -0.07 (-0.25, 0.11) |

**Supplementary Figure 4:** **Pooled prevalence of diabetes among TB patients in India by region**

**Supplementary Table 5: Detailed Characteristics of the included studies assessing the impact of diabetes on TB treatment outcome in South Asia**

| **Author**  **Date of Publication** | **Study**  **Design** | **Data**  **Collection**  **Year** | **Male**  **%**  **(total TB cases)** | **Total sample TB without diabetes** | **Total**  **TB-Diabetes** | **Mean**  **age** | **Type of TB** | **Diabetes**  **diagnosis** | **Outcome** | **Confounders** | **Exclusion criteria** |
| --- | --- | --- | --- | --- | --- | --- | --- | --- | --- | --- | --- |
| Banurekha et.al, 2007^66^ | Retrospective cohort | 1998-2002  India | 77.7% | 190 | 92 | 48 | Pulmonary Tuberculosis | FPG and HBA1C | Culture conversion | none | Patients in the moribund state or with major systemic illness, abnormal biochemical profile |
| Mundra et.al, 2017^67^ | Retrospective cohort | 2014  India | 63% | 187 | 12 | 35 | Pulmonary Tuberculosis (PTB) and Extra Pulmonary Tuberculosis (EPTB) | not mentioned anything but 61% were unknown about diabetes status | death HR | age, sex, site of illness, category of treatment initiation, HIV status | NR |
| Nandakumar et.al, 2013^38^ | Retrospective cohort | 2010-11  India | 67.9%  79% (for diabetic) | 2127 | 667 | 46 | Undifferentiated TB | anti-diabetic drugs, FPG, RPG, postprandial blood sugar verified by records) | death, shift to MDR TB treatment, failure | age group, sex, site, and type of TB, smear result, and HIV status. | transfer in cases and below age 14 |
| Sangral et.al, 2012^47^ | Retrospective cohort | 2009-10  India | 183 (65.3%)  65.2% (for diabetic) | 257 | 23 | 54 (male) and 49(female) | Undifferentiated TB | Record,  RPG, OGTT | death, failure | NR | NR |
| Siddiqui et,al, 2016^68^ | Prospective cohort | 2014  India | 44.6% | 150 | 36 | 44 | PTB &EPTB | FPG and OGTT | death, failure. Shifted to Multi-Drug Resistant TB (MDR-TB) | None | below 15 yrs, HIV infections, medical illness, coronary artery diseases, thyroid, neurological diseases. |
| Subash et.al,2003^57^ | Retrospective cohort | 1999  India | 77% | 361 | 72 | TB-diabetes 49.5 (TB only NR) | Undifferentiated TB | Case record, FPG | MDR-TB | none | Less than 12 years of age |
| Velayutham et.al, 2018^63^ | Prospective cohort | NR  India | 72% | 885 | 223 | 41 | Pulmonary TB | capillary blood glucose | Recurrence | none | Patients not initiated-Anti tuberculosis treatment, <18 age, smear result not available and refused consent |
| Viswanath et.al, 2014^69^ | Retrospective cohort | 2011  India | 74.3%  76% (  for diabetic) | 149 | 96 | TB-diabetes 49.9 TB only 37.5 | Pulmonary TB | FPG, HBA1C | death, failure. Shifted to MDR | None | TB patients with prediabetes |
| Viswanath et.al, 2016^65^ | Prospective cohort | 2012  India | 76.1%  80%) diabetic male) | 120 | 89 | TB-diabetes 50; TB :48 | Undifferentiated TB | OGTT | Death, Failure | age, sex, smoking, alcohol, adherence | HIV, impaired glucose test or impaired fasting glucose on OGTT |

#NR: Not Reported

FPG: Fasting plasma glucose

RPG: Random plasma glucose

#2H-OGTT: 2h Oral Glucose Tolerance Test

#EPTB: Extra Pulmonary Tuberculosis

HBA1C: glycosylated hemoglobin

**Supplementary table 6: Bias Analysis of the studies included in the meta-analysis for assessing the impact of diabetes on tuberculosis treatment outcomes**

| Author | Sample selection | | | | Comparability | Outcome | | | Total Quality Score |
| --- | --- | --- | --- | --- | --- | --- | --- | --- | --- |
|  | **Representativeness of the exposed cohort** | **Selection of non-exposed cohort** | **Assessment of the Diabetes** | **Demonstration that the outcome of interest was not present at the start of the study** |  | **Assessment of the outcome** | **Was follow-up long enough for outcomes to occur** | **Adequacy of follow up of cohorts** |  |
| Banu Rekha et.al ,2007 | B* | A* | A* | A* | C | A* | B | D | 5 |
| Mundra et.al, 2017 | D | A* | D | A* | A** | B* | A* | B* | 7 |
| Nandakumar et.al,2013 | B* | A* | A* | A* | A** | B* | C | B* | 8 |
| Sangral et.al, 2012 | B* | A* | A* | A* | C | B* | A* | B* | 7 |
| Siddiqui et.al, 2016 | B* | A* | A* | A* | C | B* | A* | B* | 7 |
| Subash et.al,2003 | B* | A* | A* | A* | C | B* | A* | D | 6 |
| Velayutham et.al,2018 | B* | A* | A* | A* | C | B* | A* | B* | 7 |
| Viswanath et.al, 2014 | A* | A* | A* | A* | C | B* | A* | B* | 7 |
| Viswanah et.al, 2014 | A* | A* | A* | A* | A** | B* | A* | B* | 9 |

**References**

1 Achanta, S. *et al.* Screening tuberculosis patients for diabetes in a tribal area in South India. *Public Health Action* **3**, S43-S47 (2013).

2 Adwani, S., Desai, U. D. & Joshi, J. M. Prevalence of pre-extensively drug-resistant tuberculosis (Pre XDR-TB) and extensively drug-resistant tuberculosis (XDR-TB) among pulmonary multidrug resistant tuberculosis (MDR-TB) at a tertiary care center in Mumbai. *JKIMSU* **5**, 13-19 (2016).

3 Aftab, H. *et al.* High prevalence of diabetes and anthropometric heterogeneity among tuberculosis patients in Pakistan. *Trop Med Int Health* **22**, 465-473 (2017).

4 Agarwal, A. K., Gupta, G., Marskole, P. & Agarwal, A. A study of the patients suffering from tuberculosis and tuberculosis-diabetes comorbidity in Revised National Tuberculosis Control Program Centers of Northern Madhya Pradesh, India. *Indian J Endocrinol Metab* **21**, 570-576 (2017).

5 Alfarisi, O. *et al.* Effect of Diabetes Mellitus on the Pharmacokinetics and Pharmacodynamics of Tuberculosis Treatment. *Antimicrob Agents Chemother* **62**, doi:10.1128/aac.01383-18 (2018).

6 Balakrishnan, S. *et al.* High Diabetes Prevalence among Tuberculosis Cases in Kerala, India. *PLoS One* **7** (2012).

7 Banurekha, V. *et al.* Sputum Conversion and Treatment Success among Tuberculosis Patients with Diabetes Treated under the Tuberculosis Control Programme in an Urban Setting in South India. *Indian J Community Med* **42**, 180-182, doi:10.4103/ijcm.IJCM_179_16 (2017).

8 Christopher, D. J. *et al.* Burden of diabetes among patients with tuberculosis: 10-year experience from a tertiary care referral teaching hospital in South India. *Lung India* **37**, 232 (2020).

9 Das, S. *et al.* Bi-directional screening of tuberculosis patients for type 2 diabetes mellitus and diabetes patients for tuberculosis in Bhubaneswar, Odisha. *Int J Community Med Public Health* **4**, 2435-2442 (2017).

10 Dave, P. *et al.* Screening patients with tuberculosis for diabetes mellitus in Gujarat, India. *Public Health Action* **3**, S29-S33, doi:10.5588/pha.13.0027 (2013).

11 Duraisamy, K. *et al.* Does Alcohol consumption during multidrug-resistant tuberculosis treatment affect outcome?. A population-based study in Kerala, India. *Ann Am Thorac Soc* **11**, 712-718, doi:10.1513/AnnalsATS.201312-447OC (2014).

12 Gupta, S., Shenoy, V. P., Mukhopadhyay, C., Bairy, I. & Muralidharan, S. Role of risk factors and socio-economic status in pulmonary tuberculosis: A search for the root cause in patients in a tertiary care hospital, South India. *Trop Med Int Health* **16**, 74-78 (2011).

13 Gupte, A. N. *et al.* Trends in HbA1c levels and implications for diabetes screening in tuberculosis cases undergoing treatment in India. *Int J Tuberc Lung Dis* **22**, 800-806, doi:10.5588/ijtld.18.0026 (2018).

14 Hafeez, R., Khan, S. A., Mujahid, A. & Irshad, A. Screening of diabetes and HIV infection in newly diagnosed pulmonary tuberculosis patients. *Medical Forum Mon* **29**, 51-55 (2018).

15 Hameed, S., Zuberi, F. F., Hussain, S. & Ali, S. K. Risk factors for mortality among inpatients with smear positive pulmonary tuberculosis. *Pak J Med Sci* **35**, 1361-1365 (2019).

16 Screening of patients with diabetes mellitus for tuberculosis in India. *Trop Med Int Health* **18**, 646-654, doi:10.1111/tmi.12083 (2013).

17 Jabbar, A., Hussain, S. F. & Khan, A. A. Clinical characteristics of pulmonary tuberculosis in adult Pakistani patients with co-existing diabetes mellitus. *East Mediterr Health J* **12**, 522-527 (2006).

18 Jain, S. *et al.* Socio-economical and Clinico-Radiological Profile of 474 MDR TB Cases of a Rural Medical College. *J Assoc Physicians India* **66**, 14-18 (2018).

19 Jali, M. V. *et al.* Diabetes mellitus and smoking among tuberculosis patients in a tertiary care centre in Karnataka, India. *Public Health Action* **3**, S51-S53 (2013).

20 Jawad, F., Shera, A. S., Memon, R. & Ansari, G. Glucose intolerance in pulmonary tuberculosis. *J Pak Med Assoc* **45**, 237-238 (1995).

21 Khanna, A., Lohya, S., Sharath, B. & Harries, A. Characteristics and treatment response in patients with tuberculosis and diabetes mellitus in New Delhi, India. *Public Health Action* **3**, 48-50 (2013).

22 Kornfeld, H. *et al.* Impact of Diabetes and Low Body Mass Index on Tuberculosis Treatment Outcomes. *Clinical Infectious Diseases* (2020).

23 Kottarath, M. D., Mavila, R., Achuthan, V. & Nair, S. Prevalence of diabetes mellitus in tuberculosis patients: a hospital based study. *Int J Res Med Sci.* **3**, 2810-2814 (2015).

24 Kubiak, R. W. *et al.* Interaction of nutritional status and diabetes on active and latent tuberculosis: A cross-sectional analysis. *BMC Infect Dis* **19** (2019).

25 Kumpatla, S., Aravindalochanan, V., Rajan, R., Viswanathan, V. & Kapur, A. Evaluation of performance of A1c and FPG tests for screening newly diagnosed diabetes defined by an OGTT among tuberculosis patients-A study from India. *Diabetes Res Clin Pract* **102**, 60-64 (2013).

26 Latif, A. *et al.* Did diabetes mellitus affect treatment outcome in drug-resistant tuberculosis Patients in Pakistan from 2010 to 2014? *Public Health Action* **8**, 14-19 (2018).

27 Lisha, P., James, P. & Ravindran, C. Morbidity and mortality at five years after initiating Category I treatment among patients with new sputum smear positive pulmonary tuberculosis. *Indian J Tuberc* **59**, 83-91 (2012).

28 Mahato, R. K., Laohasiriwong, W. & Koju, R. The role of type 2 diabetes mellitus on the clinical manifestation of pulmonary tuberculosis: A study from Nepal. *J Clin Diagn Res* **13**, LC09-LC14 (2019).

29 Marak, B., Kaur, P., Rao, S. R. & Selvaraju, S. Non-communicable disease comorbidities and risk factors among tuberculosis patients, Meghalaya, India. *Indian J Tuberc* **63**, 123-125 (2016).

30 Manjareeka, M., Palo, S. K., Swain, S., Pati, S. & Pati, S. Diabetes Mellitus among Newly Diagnosed Tuberculosis Patients in Tribal Odisha: An Exploratory Study. *J Clin Diagn Res* **10**, Lc06-lc08, doi:10.7860/jcdr/2016/20999.8704 (2016).

31 Mave, V. *et al.* Prevalence of dysglycemia and clinical presentation of pulmonary tuberculosis in Western India. *Int J Tuberc Lung Dis* **21**, 1280-1287, doi:10.5588/ijtld.17.0474 (2017).

32 Mallikarjuna Reddy, C., Jahnavi, K. & Swetha Madas, H. B. M. association of type II diabetes mellitus with pulmonary tuberculosis: a clinical and radiological study. *Int J Adv Med* **2**, 375-378 (2015).

33 Mehta, S., Yu, E. A., Ahamed, S. F., Bonam, W. & Kenneth, J. Rifampin resistance and diabetes mellitus in a cross-sectional study of adult patients in rural South India. *BMC infectious diseases* **15**, 451-451, doi:10.1186/s12879-015-1204-5 (2015).

34 Mukhtar, F. & Butt, Z. A. Risk of adverse treatment outcomes among new pulmonary TB patients co-infected with diabetes in Pakistan: A prospective cohort study. *PLoS One* **13** (2018).

35 Nagar, V. *et al.* A study to assess the blood glucose level among diagnosed cases of tuberculosis registered at a tuberculosis unit of Bhopal city, Madhya Pradesh, India. *Int J Med Sci Public Health* **4**, 245-249 (2015).

36 Naik, B. *et al.* Is screening for diabetes among tuberculosis patients feasible at the field level? *Public Health Action* **3**, S34-S37 (2013).

37 Nair, S. *et al.* High prevalence of undiagnosed diabetes among tuberculosis patients in peripheral health facilities in Kerala. *Public Health Action* **3**, S38-S42 (2013).

38 Kv, N. *et al.* Outcome of Tuberculosis Treatment in Patients with Diabetes Mellitus Treated in the Revised National Tuberculosis Control Programme in Malappuram District, Kerala, India. *PLoS One* **8** (2013).

39 Nandasena, S., Senavirathna, C., Munasinghe, C., Wijesena, C. & Sucharitharathna, R. Characteristics and sputum conversion of tuberculosis (TB) patients in Kalutara, Sri Lanka. *Indian J Tuberc* **66**, 76-80, doi:10.1016/j.ijtb.2018.04.008 (2019).

40 Padmalatha, P. & Hema, K. Study on prevalence of diabetes mellitus in tuberculosis patients attending a tertiary care hospital in Guntur, Andhra Pradesh. *Indian J Basic Appl Med Res* **4**, 494-498 (2014).

41 Pande, T. *et al.* Prevalence of diabetes mellitus amongst hospitalized tuberculosis patients at an Indian tertiary care center: A descriptive analysis. *PLoS One* **13** (2018).

42 Prakash, B. C. *et al.* Tuberculosis-diabetes mellitus bidirectional screening at a tertiary care centre, south india. *Public Health Action* **3**, S18-S22 (2013).

43 Raghuraman, S., Vasudevan, K. P., Govindarajan, S., Chinnakali, P. & Panigrahi, K. C. Prevalence of Diabetes Mellitus among Tuberculosis Patients in Urban Puducherry. *N Am J Med Sci* **6**, 30-34, doi:10.4103/1947-2714.125863 (2014).

44 Rajapakshe, W. *et al.* Screening patients with tuberculosis for diabetes mellitus in Ampara, Sri Lanka. *Public Health Action* **5**, 150-152 (2015).

45 Rawat, J., Sindhwani, G. & Biswas, D. Effect of age on presentation with diabetes: Comparison of nondiabetic patients with new smear-positive pulmonary tuberculosis patients. *Lung India* **28**, 187 (2011).

46 Rifat, M. *et al.* Development of multidrug resistant tuberculosis in Bangladesh: A case-control study on risk factors. *PLoS One* **9** (2014).

47 Sangral, R., Kumar, D. & Bhatia, A. S. Diabetes mellitus among tuberculosis patients in a rural population of Jammu-a community based observational study. *JK science* **14**, 177 (2012).

48 Sarker, M. *et al.* Double trouble: Prevalence and factors associated with tuberculosis and diabetes comorbidity in Bangladesh. *PLoS One* **11** (2016).

49 Sarvamangala, K. & Banerjee, A. Comparative study of type II diabetes mellitus and HIV co-morbidity among tuberculosis patients attending tertiary care hospital in davangere. *Indian J Public Health Res Dev* **5**, 192-197 (2014).

50 Shahi, R. K. Presentation of pulmonary tuberculosis with or without co-existing type 2 diabetes mellitus—a prospective study. *Clin Diabetes* **5**, 159-163 (2016).

51 Sharma, D. *et al.* Prevalence of Diabetes Mellitus and its Predictors among Tuberculosis Patients Currently on Treatment. *Indian J Community Med* **43**, 302-306, doi:10.4103/ijcm.IJCM_230_18 (2018).

52 Sharma, B., Khanal, V. K., Jha, N., Pyakurel, P. & Gurung, G. N. Study of the magnitude of diabetes and its associated risk factors among the tuberculosis patients of Morang, Eastern Nepal. *BMC public health* **19**, 1545-1545, doi:10.1186/s12889-019-7891-x (2019).

53 Shivaramakrishna, H., Gangadharan, P. & Murali, L. Prevalence and Risk Factors for Diabetes Mellitus among Tuberculosis Patients-A Study in Tamil Nadu. *Indian J Public Health Res Dev* **7**, 258-263 (2016).

54 Siddiqui, A. N., Khayyam, K. U., Siddiqui, N., Sarin, R. & Sharma, M. Diabetes prevalence and its impact on health-related quality of life in tuberculosis patients. *Trop Med Int Health* **22**, 1394-1404 (2017).

55 Singhi, L. *et al.* Non-response to first-line anti-tuberculosis treatment in Sikkim, India: A risk-factor analysis study. *Public Health Action* **8**, 162-168 (2018).

56 Sreeramareddy, C. T., Panduru, K. V., Verma, S. C., Joshi, H. S. & Bates, M. N. Comparison of pulmonary and extrapulmonary tuberculosis in Nepal - A hospital-based retrospective study. *BMC Infectious Diseases* **8 (no pagination)** (2008).

57 Subhash, H. S. *et al.* Drug resistant tuberculosis in diabetes mellitus: A retrospective study from south India. *Trop Doct* **33**, 154-156 (2003).

58 Tabassum MN, G. A., Tabassum S, Sheikh UM, Noor S, Ikram N. . Determination of risk factors among Tuberculosis patients at public sector Hospital Lahore. . *PJMHS* **13**, 792-795 (2019).

59 Tahir, Z. *et al.* Diabetes mellitus among tuberculosis patients: a cross sectional study from Pakistan. *Afr Health Sci* **16**, 671-676, doi:10.4314/ahs.v16i3.5 (2016).

60 Thapa, B., Paudel, R., Thapa, P., Shrestha, A. & Poudyal, A. Prevalence of diabetes among tuberculosis patients and associated risk factors in Kathmandu valley. *STAC* **12**, 20-27 (2015).

61 Tiwari, V. K., Verma, P. & Raj, S. Factors associated with Diabetes Mellitus among Tuberculosis Patients attending Tertiary Care Hospital in Delhi, India. *Indian J Comm Health* **28** (2016).

62 Usmani, R. A. *et al.* Diabetes mellitus among tuberculosis patients in a tertiary care hospital of Lahore. *J Ayub Med Coll Abbottabad* **26**, 61-63 (2014).

63 Velayutham, B. *et al.* Recurrence of tuberculosis among newly diagnosed sputum positive pulmonary tuberculosis patients treated under the Revised National Tuberculosis Control Programme, India: A multi-centric prospective study. *PLoS One* **13**, e0200150, doi:10.1371/journal.pone.0200150 (2018).

64 Viswanathan, V. *et al.* Prevalence of diabetes and prediabetes and associated risk factors among tuberculosis subjects in India. *Diabetes* **61**, A382 (2012).

65 Viswanathan, A. A. & Gawde, N. C. Effect of type II diabetes mellitus on treatment outcomes of tuberculosis. *Lung India: official organ of Indian Chest Society* **31**, 244 (2014).

66 Banu Rekha, V. V. *et al.* Sputum conversion at the end of intensive phase of Category-1 regimen in the treatment of pulmonary tuberculosis patients with diabetes mellitus or HIV infection: An analysis of risk factors. *Indian J Med Res* **126**, 452-458 (2007).

67 Mundra, A., Deshmukh, P. R. & Dawale, A. Magnitude and determinants of adverse treatment outcomes among tuberculosis patients registered under Revised National Tuberculosis Control Program in a Tuberculosis Unit, Wardha, Central India: A record-based cohort study. *J Epidemiol Glob Health* **7**, 111-118, doi:10.1016/j.jegh.2017.02.002 (2017).

68 Siddiqui, A. N., Khayyam, K. U. & Sharma, M. Effect of Diabetes Mellitus on Tuberculosis Treatment Outcome and Adverse Reactions in Patients Receiving Directly Observed Treatment Strategy in India: A Prospective Study. *Biomed Res. Int* **2016 (no pagination)** (2016).

69 Viswanathan, V. *et al.* Effect of diabetes on treatment outcome of smear-positive pulmonary tuberculosis--a report from South India. *J Diabetes Complications* **28**, 162-165, doi:10.1016/j.jdiacomp.2013.12.003 (2014).
